# Determinants of Iron Deficiency Anemia in a Cohort of Children Aged 6-71 Months Living in the Northeast of Minas Gerais, Brazil

**DOI:** 10.1371/journal.pone.0139555

**Published:** 2015-10-07

**Authors:** Francisca Helena Calheiros Zanin, Camilo Adalton Mariano da Silva, Élido Bonomo, Romero Alves Teixeira, Cíntia Aparecida de Jesus Pereira, Karina Benatti dos Santos, Maria Arlene Fausto, Deborah Aparecida Negrão-Correa, Joel Alves Lamounier, Mariângela Carneiro

**Affiliations:** 1 Departamento de Parasitologia, Instituto Ciências Biológicas, Universidade Federal de Minas Gerais, Campus Pampulha, Belo Horizonte, 31270–901, MG, Brazil; 2 Departamento de Nutrição Clínica e Social, Escola de Nutrição, Universidade Federal de Ouro Preto, Campus Universitário, Ouro Preto, 35400–000, MG, Brazil; 3 Faculdade de Ciências Biológicas e da Saúde, Universidade Federal dos Vales do Jequitinhonha e Mucuri, Campus JK, Diamantina, 39100–000, MG, Brazil; 4 Departamento de Alimentos, Escola de Nutrição, Universidade Federal de Ouro Preto, Ouro Preto, Minas Gerais, 35400–000, Brazil; 5 Universidade Federal de São João Del-Rei, Campus Centro-Oeste, Divinopolis, 36307–352, MG, Brazil; 6 Pós-Graduação em Infectologia e Medicina Tropical, Faculdade de Medicina, Universidade Federal de Minas Gerais, Belo Horizonte, Minas Gerais, Brazil; Oklahoma State University, UNITED STATES

## Abstract

Iron deficiency anemia is one of the most common nutritional disorders worldwide. The aim was to identify the prevalence and incidence of anemia in children and to identify predictors of this condition, including intestinal parasites, social, nutritional and environmental factors, and comorbidities. A population-based cohort study was conducted in a sample of 414 children aged 6–71 months living in Novo Cruzeiro in the Minas Gerais State. Data were collected in 2008 and 2009 by interview and included socio-economic and demographic information about the children and their families. Blood samples were collected for testing of hemoglobin, ferritin and C-reactive protein. Anthropometric measurements and parasitological analyses of fecal samples were performed. To identify risk factors associated with anemia multivariate analyses were performed using the generalized estimating equations (GEE). In 2008 and 2009, respectively, the prevalence rates of anemia were 35.9% (95%CI 31.2–40.8) and 9.8% (95%CI 7.2–12.9), the prevalence rates of iron deficiency were 18.4% (95%CI 14.7–22.6) and 21.8% (95%CI 17.8–26.2), and the incidence rates of anemia and iron deficiency were 3.2% and 21.8%. The following risk factors associated with anemia were: iron deficiency (OR = 3.2; 95%CI 2.0-.5.3), parasitic infections (OR = 1.9; 95%CI 1.2–2.8), being of risk of or being a low length/height-for-age (OR = 2.1; 95%CI 1.4–3.2), and lower retinol intake (OR = 1.7; 95%CI 1.1–2.7), adjusted over time. Nutritional factors, parasitic infections and chronic malnutrition were identified as risk factors for anemia. These factors can be verified in a chronic process and have been classically described as risk factors for these conditions.

## Introduction

The etiology of anemia is multifactorial. In clinical terms anemia is an insufficient mass of Red Blood Cells circulating in the blood; in public health terms anemia is defined as a hemoglobin concentration below the established cut-off levels [1]. Global estimates show that 50% of anemia cases are due to iron deficiency, a condition known as iron deficiency anemia. Anemia is a common public health problem in individuals from developed and developing countries, with the main risk factors being low-iron diets, low iron absorption due to the presence of phytates and phenolic compounds in the diet, and life periods characterized by a high nutritional demand such as pregnancy or growth spurts [2–4]. Other risk factors, such insufficient folate or vitamin A intake, inflammatory and infectious processes (especially malaria and infections with parasites of the Ancylostomidae family) can also cause anemia. Inherited anemia, such as hemoglobinopathies and other genetic deficiencies related to enzyme production, and types of anemia that result from congenital or acquired immunologic abnormalities [5–7] also contributing to the prevalence of anemia in many populations.

In Brazil, the National Demographic and Health Survey of Children and Women (Pesquisa Nacional de Demografia e Saúde da Criança e da Mulher—PNDS, 2006) [8], indicates a prevalence of anemia of 20.9% among children aged between 6 and 59 months. However, an unequal regional distribution can be observed, with anemia prevalence rates that vary from 10.4% to 25.5%, respectively, in the Northern and Northeastern areas of the country [8].

In recent years, some studies were conducted in the Minas Gerais State to estimate the prevalence of anemia in children. The prevalence rates found in these studies were as follows: 30.6% among children aged 6–18 months [9]; 22.6% among children aged 6–84 months [10]; 36.2% among children aged 6–71 months [11]; and 30.8% and 38.3% among children who attended kindergarten [12,13] However, few population-based studies have evaluated risk factors for anemia in children.

Epidemiologic population-based studies that identify the determinants of anemia can contribute to identifying interventions to improve the quality of life among the population of low-income regions of Brazil. The objective of this study was to evaluate the prevalence and incidence rates of anemia in children aged 6–71 months and to estimate the association of environmental and individual factors with anemia in this age group.

## Methods

### Ethical considerations

This study was approved by the Research Ethics Committees of the Federal University of Minas Gerais (No. 184/2006; No. 255/2008). Legal guardians of the children involved in this study were required to sign an Informed Consent Form at the enrollment and follow-up phase. The results were sent to the Municipality Health Services of Novo Cruzeiro to ensure the administration of adequate treatment when needed.

### Population and study design

A population-based cohort study included children aged 6–71 months was carried out from 2008 to 2009 in the city of Novo Cruzeiro (Human Development Index (HDI): 0.571), which is located in the Jequitinhonha Valley region, Minas Gerais State [14]. This city had a population of 30725 inhabitants in 2010, with a population density of 18.04 inhabitants/Km^2^; 70% of the population lived in rural areas [15]. This municipality is assisted by primary care programs such as the Family Health Program (FHP), a strategy currently adopted by the Brazilian Unified Health System (Sistema Único de Saúde—SUS] that assist more than 90% of Novo Cruzeiro’s population [16].

This study is part of a broader epidemiological research project that evaluated child health among residents of the Jequitinhonha Valley, Northeast Minas Gerais State, Brazil. The sample calculation and sampling process were previously described by Macedo *et al*. (2012) [17]. Briefly, the following parameters were used to estimate the sample size: 2718 children aged 6–71 months were living in Novo Cruzeiro [15]; anemia prevalence rate of 36.2% [11]; the estimated precision was 5%, the effect design was 1.5, and the confidence interval was 95%. A total of 439 children were included in the baseline phase, 414 of whom were evaluated in the follow-up phase.

The sampling unit was the household, which was identified through the municipal census conducted by the Family Health Program (FHP). The number of households selected in rural and urban regions was proportional to the existing number of households in each region. The following two-stage sampling scheme was used: (1) simple random sample of communities assisted by the FHP was selected; (2) households were chosen randomly within each selected community. Additionally, proportionality criteria based in children number were adopted to select children in each selected community. All children aged 6 to 71 months living in the selected households were invited to participate in the study.

### Data collection

The baseline and the follow-up phase were conducted on March, 2008 and July, 2009. In both phases, the data collections were performed by a trained staff and the data collection were performed at children's homes (baseline) and school or health centers (follow-up).

The variables collected at the baseline were on the following factors: (i) demographic and child-related questions (e.g., sex, age, race, weight, height, food intake, reported morbidities, access to health service), (ii) parental socioeconomic status (e.g., educational level, income, schooling, employment, number of family members, number of children), (iii) household characteristics (e.g., walls, roof and floor construction materials, number of rooms), and (iv) environmental conditions (e.g., water quality, access to sanitation and public water, sewage availability, domestic refuse storage and disposal).

In the follow-up stage, the variables collected were related to anemia (previous history of individual and familiar anemia, treatment), comorbidities, parasitic infections (signs and symptoms suggestive of a recent infection), and the use of anthelmintics or nutritional supplements (ferrous sulfate, multivitamins, vitamin A or others).

In both phases anthropometric data (weight and height) and biological samples (venous blood and fecal samples) were collected using the same procedure.

### Blood samples analyses

Blood samples were used for testing of hemoglobin (Hb), ferritin, C-reactive protein (CRP). Two venous blood samples were obtained in a closed blood collection system (Monovette®) with EDTA and without anticoagulant.

In the baseline, Hb measurement was obtained in the field with the HemoCue® system [18], and ferritin serum levels (chemiluminescence) and CRP (nephelometry) were measured at the Central Clinical Laboratory of the Clinical Hospital of the Federal University of Minas Gerais (UFMG). In the follow-up phase, a complete hemogram was conducted (using an automatic counter made by either Cobas® 60 or Roche®; the thick blood film was stained with May-Grunwald Giemsa hematological stains) in a clinical laboratory that was located in the region of data collection. Ferritin serum levels (Access Ferritin) and CRP (Siemens® LKCRP/Immulite) were analyzed in the Clinical Analyses Laboratory (LAPAC) of the Pharmacy School of the Federal University of Ouro Preto (UFOP). Although Hb was analyzed using different methods in the two phases of the study (HemoCue® vs hemogram), significant differences were not expected in the results due to the methodology used [19].

Anemia was defined as Hb<11.0 g/dL for children aged 6–59.9 months and Hb <11.5 g/dL for children aged ≥60 months. Anemia was classified into the following categories: severe anemia if Hb was < 7.0 g/dL, moderate anemia if Hb ranged between 7.0 and 9.0 g/dL, and mild anemia if was Hb > 9.0 g/dL and < 11.0 g/dL [20].

Iron deficiency was diagnosed if ferritin was <12 μg/L for children aged between 6 and 60 months; in older children, iron deficiency was diagnosed by a ferritin level <15 μg/L or a ferritin level <30 μg/L in a child with a CRP ≥10 mg/L [20].

### Parasitological exams

Two fecal samples were obtained. One was collected with a preservative—buffered formalin solution (10% formaldehyde in a buffered saline solution (PBS) containing 13.7 mM of NaCl, 0.27 mM of KCl, 0.14 mM of KH_2_SO_4_ and 0.43 mM of Na_2_HPO_4_.7H_2_O); the other sample was collected without preservatives. In both phases, the collected fecal samples were processed using the methods of Hoffman, Pons and Janer—HPJ (1934) [21] and Kato-Katz [22]. The collected preservative samples were sent to LAPAC/UFOP and to the Laboratory for Helminthic Immunology at the Parasitology Department (UFMG). The parasitological results were considered independently of the method used.

### Test reliability for fecal and blood analyses

Two independent laboratory technicians analyzed the HPJ samples. Duplicates of 10% of the HPJ samples were created using the Kato-Katz method; each duplicate received a different number from the original sample and was masked for re-examination. Biochemical and immunological analyses were repeated whenever the values were doubtful.

### Anthropometric data and food intake

Weight was measured using a scale with a sensitivity of 50 grams and a capacity of 150 kg. The smallest children were weighed in the arms of an adult who had been previously weighed. Height was measured using a wooden anthropometer was used with a ruler measuring up to 2000 mm. Children's length up to 24 months old were measured in a recumbent position.

Weight-for-age, length/height-for-age, weight-for-length/height and body mass index-for-age z-scores values were generated for children aged 6–60 months, using the Anthro software; and were also generated for children aged>60 months, using the Anthro Plus software (http://www.who.int/childgrowth/software/en/). Body mass index (BMI)-for-age z-scores were used to evaluate the anthropometric status of children older than 24 months. [23]

The criteria used for assessing nutritional status of the children based on the z-scores of the anthropometric data were: normal (z-score ≥ −1 and ≤2); nutritional deficit (z-score <−2); nutritional risk (z-score ≥−2 and <−1); and nutritional excess (z-score >2) [24].

Information on children’s food intake was only obtained in the baseline using the Semi-Quantitative Food Frequency Questionnaire (SQFFQ) [25] that had previously been tested and adjusted in the Jequitinhonha Valley [11,17]. The information about children's food intake was provided by parents and/or guardians who used a photographic album of foods [26] to identify the habitual portion sizes. For children who attended kindergarten or school, the food provided by the school was weighed during three days. The average value per capita of food intake in this place was calculated and added to the per capita value obtained from the SQFFQ, providing an estimate of the child’s total food intake. National food composition tables were used to estimate the intake of carbohydrate, protein, lipids, energy, iron, zinc, retinol, thiamin and riboflavin [27,28].

The energy and the nutrients intake were categorized according to the children's median intakes (0 = >median and 1 = ≤median).

### Interventions performed

In August 2008, the results of the anemia and parasitological exams were delivered to the Municipality’s Health Service, and all the children with anemia and/or parasites received ferrous sulfate and/or anthelmintics. Anemia treatment information was not available for this study.

### Statistical analysis

The databases were created using EpiData (version 3.2, EpiData Association, Odense, Denmark); results were double-entered. The data were subsequently compared, corrected and analyzed with EpiInfo 2002 (Centers for Disease Control and Prevention, 2002) and Stata software (version 11.0, Stata Corporation, College Station, TX, USA).

To investigate if children that were lost presented the same characteristics of those children that remained in the follow-up phase, the chi-square test was used to comparing proportion; t-Student or ANOVA tests were used to compare means; and medians were compared using the Mann-Whitney and Kruskal-Wallis tests.

The prevalence rates (95% confidence intervals) were estimated considering the number of children with anemia and iron deficiency, according to definitions adopted, by the number of children examined. The incidence rates (95% confidence intervals) were estimated considering the number of children that were diagnosed with anemia and iron deficiency in 2009 among those without these conditions in 2008 evaluation.

Analyses of risk factors were carried out considering the data obtained in the baseline and follow-up phase. Variables that could explain anemia over time were selected, even though these variables had been collected in only one stage of the study. For the longitudinal evaluation of the association between the anemia and the independent variables, repetitive measures were evaluated in the two stages studied. The Generalized Estimation Equations (GEE) model, proposed by Diggle, Liang and Zeger (1994) [29] was used. For this analysis, the STATA “xtgee” function was used and odds ratios were estimated with 95% confidence intervals. Initially, the association was investigated between each variable and the response variable. The variables with a p<0.25 were selected and grouped into the following categories: demographic variables and children’s health care variables; socioeconomic variables; household characteristics; exam results; parasitic infections; anemia familiar history; comorbidities; anthropometry; and nutrient intake. Then, the association was investigated within of each group and the variables with a p< 0.10 were selected for the construction of final model. Variables with a low frequency and those with colinearity were excluded from the analysis. A step-by-step backward selection procedure was used to select the variables for the final model. Likelihood ratio tests were used to evaluate the models.

## Results

### Children evaluated

**Fig 1:**
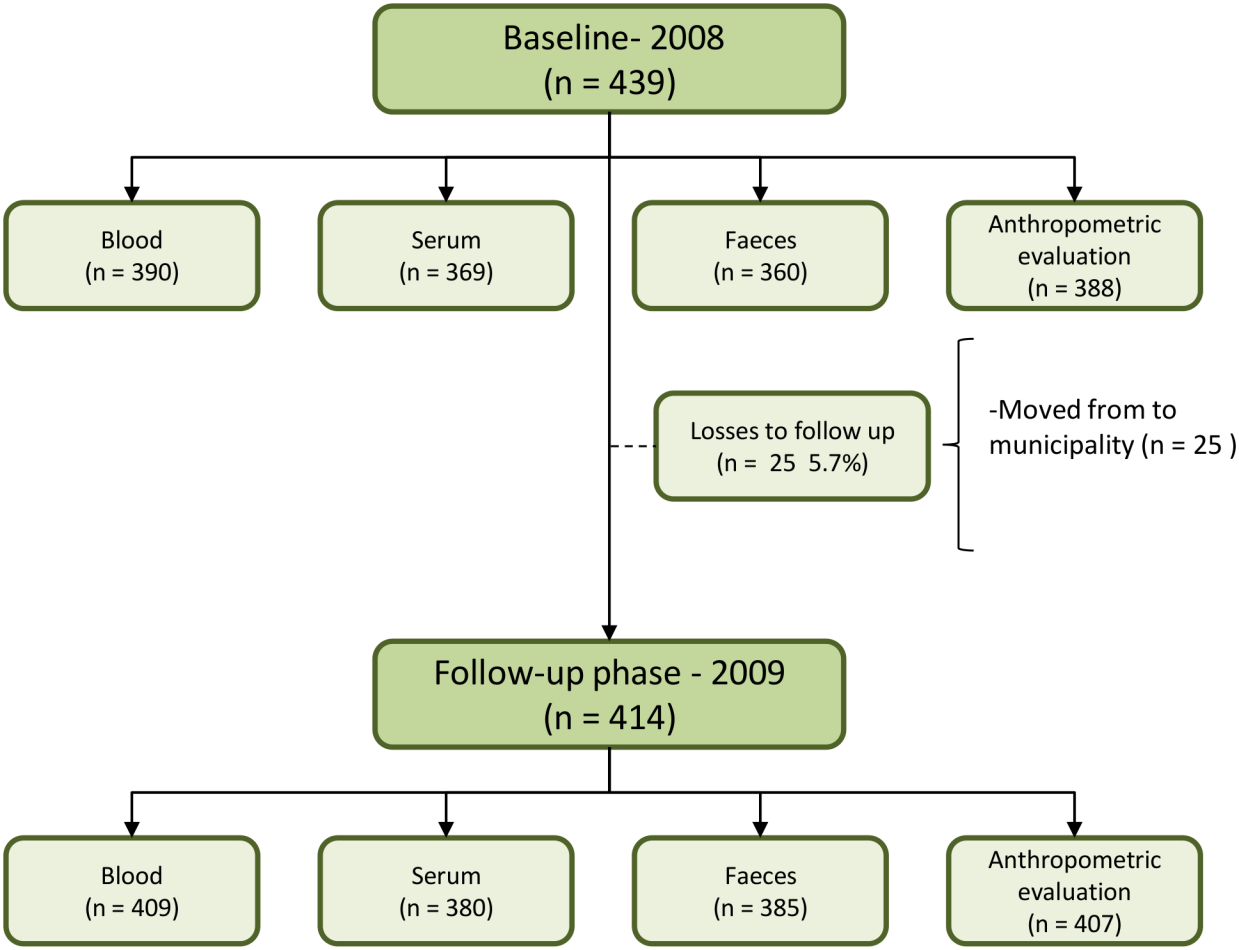
Flow diagram of the children population evaluated in two phases—2008 and 2009.

In the baseline phase (2008) were included a sample of 439 children aged between 6 and 71 months; 414 children were evaluated in the follow-up phase in 2009. There were 25 cases (5.7%) of dropout in the study; they had moved from the municipality. Furthermore, losses were also observed in biological material such as blood, serum, stool and anthropometric measurements (Fig 1). Children who participated in the follow-up stage did not differ from non-participants in terms of gender, age group, presence of parasitic infections, or iron deficiency. However, a higher proportion of participants were children of mothers with a lower educational level (p<0.005) and who lived in the rural area of the municipality (p< 0.005).

### Characterization of families, domiciles and children’s general conditions

Of the participant families, 78.8% were poor, and 52.1% lived in conditions of extreme poverty according to the United Nations Development Program [30] criteria. The majority of the families (66.8%) were registered for some projects of Brazilian Government Assistance. An average number of 5.94±2.31 residents per domicile and 2.65 ± 1.23 habitants per bedroom were observed.

Regarding the drinking water source, 29.0% of the families received water from the public network and the majority (73.1%) had treated water in their households using the filtration process (75.9%). Almost half of the households (46.3%) were without toilets, with sanitary facilities located inside or outside the houses. Only 26.2% of the households owned a sewage disposal system that was connected to a public network. The majority of the households burned their refuse (62.1%), and only 22.8% were serviced by the public waste collection system.

In the baseline, the children had a mean age (SD) of 40.9±18.8 months and 50.7% of participants were male.

Of the fecal samples examined, 41.7% in 2008 and 50.7% in 2009 were infected. In 2008, the most prevalent parasitic infections were *Schistosoma*. *mansoni* (8.6%), *Ascaris lumbricoides* (8.3%) and *Giardia duodenalis* (18.6%). Other parasites and commensal protozoans were found but had a lower prevalence. During the follow-up stage (2009), *A*. *lumbricoides* (5.7%) and *G*. *duodenalis* (15.4%) prevailed; furthermore, the *Entameba coli* commensal protozoan (21.2%) was commonly found in the analyzed samples.

### Anemia and iron deficiency

Table 1 shows the prevalence of anemia and iron deficiency globally and per age group.

**Table 1:** Anemia and iron deficiency prevalence in children evaluated by hemoglobin and ferritin levels, Novo Cruzeiro (2008 and 2009).

Variables	2008				2009			
	N	n	%	95% CI	N	n	%	95% CI
*Anemia*	390	140	35.9	(31.2; 40.8)	409	40	9.8	(7.2; 12.9)
< 36 months	157	74	47.1	(39.4; 55.0)	73	11	15.1	(8.2; 24.7)
36 to 60 months	149	36	24.2	(17.8; 31.5)	213	13	8.7	(3.4; 10.0)
> 60 months	84	30	35.7	(26.0; 46.4)	123	16	8.6	(7.9; 19.9)
*Iron deficiency*	369	68	18.4	(14.7; 22.6)	372	81	21.8	(17.8; 26.2)
< 36 months	147	45	30.6	(23.6; 38.4)	66	29	43.9	(32.4; 56.1)
36 to 60 months	143	14	9.8	(5.7; 15.5)	134	21	15.7	(10.2; 22.6)
> 60 months	79	09	11.4	(5.7; 19.9)	172	31	18.0	(12.8; 24.3)

Levels recommended by WHO (2001).

Anemia was classified as mild in 55.7% and 70.0% of the children in 2008 and 2009, respectively. Severe cases of anemia (1.4%) were only observed in 2008. Children younger than 36 months had a higher prevalence of iron deficiency and anemia. The incidence rates of anemia and an iron deficiency were 3.2% and 15.1%, respectively.

### The generalized estimation equations models (GEE)

Univariate analysis was carried out by group, taking into account all the variables, one at a time. However, Tables 2 and 3 show only the variables with P < 0.20 in univariate analyses. The variables selected in each step of the analysis and used to build the final model (Tables 2 and 3) were: iron deficiency (yes *vs*. no), infection caused by protozoans and helminthes (1 = yes *vs*. no), length/height-for-age (1 = risk or low height-for-age *vs*. 0 = normal height-for-age), carbohydrate intake (1 = ≤median *vs*. 0 = >median), retinol intake (1 = ≤median *vs*. 0 = >median), (1 = ≤median *vs*. 0 = >median), and time (0 = 2008 *vs*. 1 = 2009).

**Table 2:** Univariate analysis of longitudinal logistic regression for anemia according to socioeconomic and environmental children’s characteristics, Novo Cruzeiro, Minas Gerais, Brazil, 2008–2009.

Variables	Anemic	Non-anemic	OR (95%CI) ^(a)^	p-value
	n (%)	n (%)		
Gender				
Female	17 (42.5)	185 (50.1)	1	
Male	23 (57.5)	184 (49.9)	0.8 (0.5–0.1)	0.20
Household situation				
Urban	04 (10.0)	67 (18.2)	1	
Rural	36 (90.0)	302 (81.8)	0.7 (0.4–1.1)	0.08
Condition in the Family				
Child	37 (92.5)	333 (90.2)		
Grandchild/other relative	03 (7.5)	36 (9.8)	0.4 (0.2–0.9)	0.02
Householder has an income				
No	09 (22.5)	75 (20.4)	1	
Yes (working, retired)	31 (77.5)	293 (79.6)	0.7 (0.4–1.1)	0.13
Householder working situation				
Employed	13 (32.5)	128 (35.4)	1	
Autonomous	18 (45.0)	149 (41.2)	0.9 (0.6–1.5)	0.90
Tenant farmer, others	09 (22.5)	85 (23.5)	1.6 (0.9–2.7)	0.05
Poverty according to UNDP ^(b)^				
No	03 (8.3)	64 (20.4)	1	
Yes	33 (91.7)	250 (79.6)	0.67 (0.40–1.12)	0.124
Drinking water origin				
Public network or artesian well	17 (42.5)	124 (33.7)	1	
Shallow well or cistern	05 (12.5)	100 (27.2)	0.7 (0.4–1.1)	0.14
Dam, stream or water source	18 (45.0)	144 (39.1)	0.6 (0.4–0.9)	0.03
Sewage disposal				
Absence	55 (39.3)	109 (43.6)	1	
Public network or septic tank	32 (22.9)	38 (15.2)	1.6 (0.9–2.7)	0.09
Rudimentary septic tank	53 (37.9)	103 (41.2)	1.0 (0.7–1.6)	0.86
Iron deficiency				
No	30 (76.9)	267 (81.9)	1	
Yes	09 (23.1)	59 (18.1)	2.9 (1.9–4.5)	<0.0005
Infection				
No	34 (87.2)	314 (92.1)	1	
Yes	05 (12.8)	27 (7.9)	1.8 (1.0–3.4)	0.05
Parasitic infections				
No	11 (28.2)	92 (27.0)	1	
Yes	28 (71.8)	249 (73.0)	1.58 (1.11–2.25)	0.01
Diarrhea				
No	36 (92.3)	326 (90.1)	1	
Yes	03 (7.7)	36 (9.9)	1.54 (1.00–2.35)	0.05
Fever				
No	29 (72.5)	287 (78.0)	1	
Yes	11 (27.5)	81 (22.0)	1.32 (0.91–1.91)	0.15

^(a)^ Odds Ratio adjusted over time.

^(b)^ UNDP (The United Nations Development Programme).

**Table 3:** Univariate analysis of longitudinal logistic regression for anemia according to anthropometric and nutrient intake children’s characteristics, Novo Cruzeiro, Minas Gerais, Brazil, 2008–2009.

Variables	Anemic	Non-anemic	OR (95% CI) ^(a)^	p-value
	n (%)	n (%)		
Weight-for-height z-score				
≥ −2	23 (95.8)	188 (97.4)	1	
< −2	01 (4.2)	05 (2.6)	2.6 (0.7–9.0)	0.14
Length/Height-for-age z-score				
≥ −1	23 (57.5)	230 (62.8)	1	
< −1	17 (42.5)	136 (37.2)	1.7 (1.2–2.6)	0.006
Weight-for-age z-score				
≥ −1	29 (72.5)	262 (71.2)	1	
< −1	11 (27.5)	106 (28.8)	1.5 (1.0–2.3)	0.05
Iron intake (μg)				
> 6.63	63 (45.0)	140 (56.0)	1	
≤ 6.63	77 (55.0)	110 (44.0)	1.6 (1.1–2.4)	0.02
Energy intake (kcal)				
> 1,565	59 (42.1)	136 (54.4)	1	
≤ 1,565	81 (57.9)	114 (45.6)	1.8 (1.2–2.6)	0.005
Protein intake (g)				
> 36.9	64 (45.7)	136 (54.4)	1	
≤ 36.9	76 (56.3)	114 (45.6)	1.4 (0.9–2.1)	0.08
Lipid intake (g)				
> 51.86	63 (45.0)	134 (53.6)	1	
≤ 51.86	77 (55.0)	116 (46.4)	1.6 (1.1–2.4)	0.02
Carbohydrate intake (g)				
> 232.79	56 (40.0)	141 (56.4)	1	
≤ 232.79	84 (60.0)	109 (43.6)	2.1 (1.4–3.1)	<0.0005
Zinc intake (mg)				
> 4.66	62 (44.3)	138 (55.2)	1	
≤ 4.66	78 (55.7)	112(44.8)	1.6 (1.1–2.3)	0.03
Retinol intake (μg)				
> 369.44	60 (42.9)	139 (55.6)		
≤ 369.44	80 (57.1)	111 (44.4)	1.9 (1.3–2.9)	0.001
Thiamin intake (mg)				
> 199.62	58 (41.4)	142 (56.8)	1	
≤ 199.62	82(58.6)	108 (43.2)	1.7 (1.1–2.5)	0.01
Riboflavin intake (mg)				
> 182.0	58 (41.4)	142 (56.8)	1	
≤ 182.0	108 (43.2)	108 (43.2)	1.6 (1.1–2.4)	0.02

^(a)^ Odds Ratio adjusted over time.

The final model for anemia shows (Table 4): iron deficiency was associated with the occurrence of anemia; children with iron deficiency had a 3.2-fold higher risk of anemia than children with normal levels of ferritin. Both parasitic infection and a low retinol intake increased the risk of anemia by a factor of almost two. Children with a risk of or a low length/height-for-age had a risk for anemia twice as high as those with a normal z score. A reduced risk of anemia (OR = 0.19) was observed during the follow-up.

**Table 4:** Final generalized estimation equation model for anemia in children living in of Novo Cruzeiro, Minas Gerais, Brazil, 2008–2009.

Variables	OR	95% CI	Adj. OR^(a)^	95% CI	p-value
Iron deficiency (yes *vs*. no)	2.9	(1.9–4.5)	3.2	(2.0–5.3)	<0.0005
Parasitic infections (yes *vs*. no)	1.6	(1.1–2.3)	1.9	(1.2–2.8)	0.003
Length/Height-age z-score (risk or low *vs*. normal)	1.7	(1.2–2.6)	2.1	(1.4–3.2)	0.001
Retinol intake (lower than median *vs*. higher than median)	1.6	(1.1–2.3)	1.7	(1.1–2.7)	0.02
Time (2008 *vs*. 2009)			0.2	(0.1–0.3)	<0.0005

^(a)^ = Adjusted Odds Ratio.

## Discussion

The results of the present study showed that the prevalence rates of anemia were 35.9% (2008) and 9.8% (2009), and the incidence rate was 3.2%. Concerning serum ferritin levels the prevalence were 18.4% (2008) and 21.8% (2009) and the incidence rate was 2.4% Children under the age of 36 months had the highest rates of anemia and iron deficiency during both periods.

In this study, the observed prevalence rate of anemia in 2008 was similar to rates observed in other studies conducted in Brazil at approximately the same time [11,13, 31–33]. A reduction in the prevalence of anemia was observed between 2008 and in 2009, changing from a moderate-intensity public health problem to a mild-intensity problem [24] for the age group analyzed. This reduction may be due to the ferrous sulfate and antiparasitic treatments that were provided by the Municipality Health Services. The results of the blood samples analysis and parasitological exams of the first evaluation were delivered to the Municipality Health Service. The iron supplementation and anti helmintics treatment are provided by the Brazilian Unified Health System (Sistema Único de Saúde—SUS] and available in the Basic Unity of Health of the Municipality.

Differences in the methodology used for the diagnosis of anemia must be discussed. In 2008, hemoglobin levels were determined by Hemocue in the field; in 2009, blood counts and hemoglobin levels were obtained using an automated device. Although an evaluation of the technique was not performed in the present work, Hemocue is considered to be a highly accurate and robust method that permits verification of the quality of the analysis [7,34]. The methods were chosen based on sensitivity and specificity, as well as the feasibility of Hemocue usage in the field, particularly given the need to analyze a large number of samples and the distance from a reference laboratory, which made it difficult to use the gold standard method [19].

Even though the prevalence of iron deficiency was lower than rates that have been observed in other Brazilian studies [35,36], an increased number of cases was observed in 2009, suggesting a worsening in iron reserves as the children grow.

Risk factors associated with anemia included iron deficiency and the diagnosis of a parasitic infection, risk of or low length/height-for-age, and a retinol intake lower than the median, adjusted for time. Iron deficiency can cause anemia over the long term [3–4,20]. Although iron deficiency is a cause of anemia, it was kept in the models to allow an investigation of other anemia-associated factors adjusted for iron deficiency. Iron deficiency is a cause of anemia in disadvantaged regions, although multiple causes can exist independently or coexist with this deficiency [37]. The primary sources of iron are the diet and the recycling of senescent erythrocytes; the amount of iron is absorbed and regulated according to the organism’s needs: increased demand for iron can stimulate increased absorption. Factors such as iron bioavailability in foods, acidity, and the presence of solubilizing agents can influence intestinal absorption [38]. Although diet monotony [39] was not the object of this study, a low intake of essential nutrients such as iron could be the cause of iron deficiency. In addition, low iron bioavailability in an infant’s diet, particularly after weaning [37] should also be considered.

The presence of parasitic or commensal infections was also associated with anemia occurrence in the longitudinal model. Infections caused by helminthes, such as parasites of the Ancylostomidae family, can provoke anemia due to chronic intestinal blood loss [40,41]. Schistosomiasis can cause anemia via several mechanisms such as iron loss in the feces, splenic sequestration and destruction of erythrocytes due to splenomegaly, autoimmune hemolysis, and inflammation [42]. In addition to Helminthiasis, *G*. *duodenalis* [11,43] and the *E*. *coli* commensal can be associated with nutritional status [43] and indirectly contribute to anemia.

The reduced risk of anemia associated with an increased length/height-for-age z-score is an indirect indicator of the relationship between nutritional status and anemia. The association of the length/height-for-age indicator with anemia indicates that child growth is affected in the long run [44] and, consequently, that better growth is associated with a lower risk of anemia. The association between low retinol intake and anemia corroborates previous findings that relate vitamin A deficiency to anemia [45,46]. Several authors mention the association of vitamin A deficiency with the occurrence of anemia, and retinol supplementation has been proposed for children younger than five years with the aim of preventing this deficiency [47,48]. In Brazil, the vitamin A program consists of prophylactic supplementation with mega-doses of vitamin A in high risks areas such as the Jequitinhonha and Mucuri Valleys, Minas Gerais [48].

The variable “time” was included in the model and remained associated with a reduced risk of anemia. This effect is assumed to be causes the ferrous sulfate treatment offered to anemic children in 2008. In addition, most likely due to increased awareness of the problem of anemia in the municipality, the local health system and children’s relatives, may have adopted measures that contributed to reducing anemia in this region.

WHO experts recommend testing of hemoglobin, transferrin serum receptor and serum ferritin or fundamental iron studies to diagnose iron deficiency. These exams reflect functional impairment and a cellular need for iron and iron stores [20]. Although testing hemoglobin levels cannot detect iron deficiency due to the erythrocytes’ survival time [3], it can reliably diagnose anemia in populational studies. WHO recommends the cyanomethemoglobin method (used in analytic laboratories) and the Hemocue system [20]. In the presence of inflammatory and infectious processes, iron sequestration can be observed in reserve forms such as ferritin [40] and some iron deficient children may have been misclassified as iron deficient, even considering CRP values.

During the present study, selection and information bias could have occurred. Rates of loss to follow-up (5.7%) occurred in children who lived in the urban area and children whose mothers had higher levels of education. The loss of follow-up in urban areas was due to moving, reflecting the high mobility of urban families. Information bias could have occurred because the information obtained from interviews can be influenced by the interviewee’s relationship with the child, his or her memory, and the importance that the interviewee attaches to the questionnaire’s topic. The semi-quantitative food frequency questionnaire can overestimate food consumption, and the questionnaire about a family history of anemia had a high rate of non-responses; this finding is probably due to a lack of knowledge and information about the issues addressed. To minimize information bias, interviewers were trained and supervised. The limitations of the parasitological diagnostic methods are related to the absence of specific testing methods and to a single sample collection. These limitations may have led to an underestimation of the prevalence of *G*. *duodenalis*, due to the intermittent daily elimination in the feces of small quantities of cysts and helminthes, which also occurs in *S*. *stercoralis* and parasites of the Ancylostomidae family. However, the samples were examined in two laboratories that used various methods, increasing the diagnostic accuracy.

In summary, the factors associated with anemia were iron deficiency, the presence of infections caused by parasites or commensals, being at risk of or being low height-for-age, and a retinol intake that was lower than the median; time was associated with a reduced risk of anemia. Interventions by the Municipality Health Services and the treatment of anemic children with ferrous sulfate during the study period should be highlighted.

Providing conditions conducive to reducing the occurrence of inflammatory and infectious processes, improving nutritional status by ensuring that the intake of essential nutrients is adequate for growth, and guaranteeing wider access to health programs and services can help to lower the risk of anemia and iron deficiency, both of which can have serious consequences for the pediatric population.

## Supporting Information

S1 Checklist Cohort Checklist. (DOCX)
